# 

**Published:** 1913-11-22

**Authors:** 


### A MEDICAL DEPUTY MAYOR.

Dr. F. W. Stone has secured a triumph at the municipal elections at Dover, where he was returned for the town ward at the head of the poll, eighty-three votes ahead of his colleague, Sir Montague Bradley, and 619 ahead of their opponent, Dr. Morrison, the candidate of the party that had been dominant in Dover for twenty-two years. The bulletin posted up by the newspaper which had opposed Dr. Stone most strongly said: "The result was received with great enthusiasm." Dr. Stone is a Conservative, but has broken away from his party in the Council on questions of finance, housing, and docks on the sea front. He is comparatively a newcomer at Dover, so that his popularity seems remarkable. Dr. Stone, with a large majority, has also been chosen as Deputy-Mayor of Dover.

### NEW PRESIDENT OF BRISTOL GENERAL HOSPITAL.

At a recent meeting of the Committee of the Bristol General Hospital Mr. George Alfred Wills was unanimously elected President of that Institution. Mr. Wills is the head of the well-known tobacco firm which is the largest in the city of Bristol and one of the largest in England. Mr. Wills is no stranger, having served as Vice-President and upon the House Committee for some years.

### MANCHESTER AND DISTRICT ASSOCIATION OF HOSPITAL SECRETARIES.

At a quarterly meeting of the Manchester and District Association of Hospital Secretaries held last week—Mr. W. G. Carnt, Manchester Royal Infirmary, in the chair—the following new members were elected : Mr. Francis H. Moore, general superintendent and secretary of Liverpool Royal Infirmary; Mr. F. J. Bray, general manager of Leeds General Infirmary; and Mr. A. E. Briscoe, secretary of the Bolton Infirmary, Lancs. The annual dinner was fixed to take place at the Albion Hotel, Piccadilly, Manchester, on Saturday, January 31, 1914.

### HOSPITAL BEQUEST REVOKED.

Mr. Augustus Walter Rawcliffe, of Haigh, Lancs, brewer, wine and spirit merchant, and maltster, who left estate valued at £130,363 gross, by a codicil has revoked a bequest of £1,000 to the Chorley Dispensary and Cottage Hospital, stating that " having regard to the recent unjust and unequally distributed taxation I do not feel called upon to make any charitable legacies."

